# Bochdalek Hernia As A Cause Of Intermittent AV Block

**Published:** 2011-10-02

**Authors:** Kakhaber Etsadashvili, Haroon Mohammed Rashid, Khatuna Jalabadze, Anzor Melia

**Affiliations:** Cardiology Clinic "Guli", Tbilisi, Georgia

**Keywords:** AV block, Bochdalek hernia

## Abstract

Congenital diaphragmatic hernia is very rare cause of AV block. We report such a patient with sick sinus node syndrome and previous AAIR pacemaker implantation, in which intermittent AV block was diagnosed by 24-hours ECG monitoring and upgrade of pacing system to DDDR was suggested.

## Introduction

Bochdalek hernia makes up about 90% of all diaphragmatic hernia cases and about one in every 2200 to 12,500 births every year, with a ratio of 3:2 of males to females [1]. Bochdalek hernia can be a life-threatening condition. Between 25-60% of infants, die from a Bochdalek hernia, mostly due to respiratory insufficiency [2]. Diagnosis is usually done at early age and therefore is rare in adults.

We report a case of a 62-years old male, where Bochdalek hernia was diagnosed by the routine preoperative examination for the pacemaker implantation and was suspected as a cause of intermittent AV block due displacement and/or mechanical compression of heart.

## Case presentation

**Figure 1:**
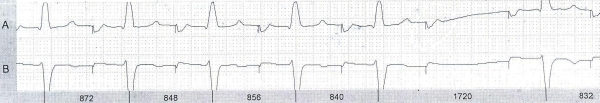
ECG 24-hours monitoring tracing. Shows proper atrial pacing and intermittent 2:1 AV block.

**Figure 2:**
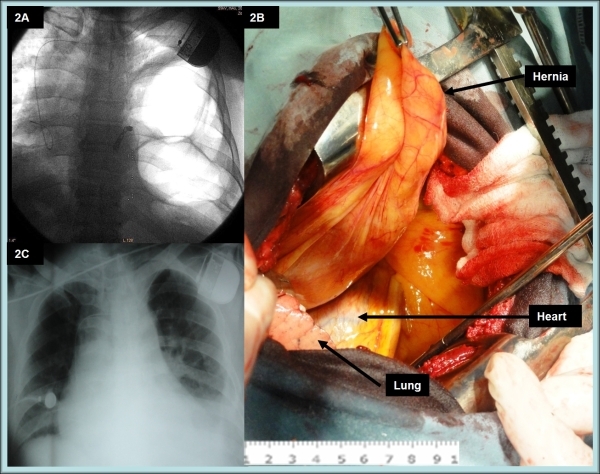
2A, Initial chest fluoroscopy shows the permanent pacemaker with the pacing lead in right atrium and hypo dense (bowel density) mass in the left chest cavity. 2B, Herniated abdominal viscera into the thorax. Heart and lung tissue are also seen after thoracotomy. 2C, Chest X-ray after surgical repair: normal anatomical position of chest organs.

A 62-years-old male was presented to our clinic because of loss of consciousness. Five years ago a single chamber AAIR pacemaker was implanted due to sick sinus node syndrome. Since then patient was symptom free, until an episode of syncope occurred recently. Echocardiography revealed preserved left ventricular function (EF=58%) without structural heart disease. 24-hours ECG showed an appropriate atrial stimulation without ST-segment/T-wave changes during the exertion. Few episodes of intermittent 2:1 AV block during rest were observed (Figure 1). Upgrade of pacing system form AAIR to DDDR was advised and the patient was sent to our clinic. Routine chest X-Ray revealed the permanent pacemaker with the pacing lead in right atrium and hypo dense (bowel density) mass in the left chest cavity with the collapse of the left lung and shift of the heart and big vessels towards the right, resulting in reduction of right lung volume (Figure 2A). Diaphragmatic hernia was assumed. Few published reports show an influence of pneumothorax on atrioventricular conduction by increasing the parasympathetic tone [3,4]. Significant increasing of vagal tone due to displacement/mechanical compression of the heart and the left lung collapse was suspected as a reason of intermittent AV block. Patient was referred to surgical department, where Bochdalek hernia was diagnosed. Back-up temporary pacemaker lead was implanted in the right ventricle and surgical correction was performed. Thoracotomy revealed defect of posterolateral diaphragmatic wall with herniated abdominal viscera into the thorax (Figure 2B). Additionally ectopic intrathoracic left kidney was found, which represents less than 5% of all renal ectopias [5]. Hernia was repaired. Post operative day one X-ray showed normal position of the chest organs (Figure 2C). Repeat 24-hours ECG monitoring after a day of operation and then 3 months later revealed the normal function of permanent pacemaker (AAIR mode) with proper atrial pacing and no sign of AV block. Stable pacing and sensing parameters, as well as normal lead impedance was detected by the pacemaker interrogation, and the mean estimated longevity was 3 years. The patient was discharged in a stable condition. The follow up period of 6 months was uneventful.

## Discussion

The relation between the diaphragmatic hernias and AV conduction disturbances is not well established so far. Moreover, we could not find any published case, where diaphragmatic hernia evoked AV block. Only few cases show the relation between the increased intrathoracic pressure (e.g. pneumothorax) and AV conduction disturbances [3,4]. The patient suffered due to syncopal episodes last time and several episodes of paroxysmal AV block were found during the 24 hours ECG monitoring prior to surgery. Absence of another cause of AV Block in our case suggests the diaphragmal hernia might be the only cause of intermittent AV block and syncope. After surgical intervention we could not reveal any episodes of AV block as well as the patient stays free of syncope during the follow-up period that suggests connection of AV conduction disturbance to hernia.

## Conclusion

This case illustrates that Bochdalek hernia could be a cause of intermittent AV block, due to displacement and/or mechanical compression of the heart.
